# Inhibiting Delta-6 Desaturase Activity Suppresses Tumor Growth in Mice

**DOI:** 10.1371/journal.pone.0047567

**Published:** 2012-10-24

**Authors:** Chengwei He, Xiying Qu, Jianbo Wan, Rong Rong, Lili Huang, Chun Cai, Keyuan Zhou, Yan Gu, Steven Y. Qian, Jing X. Kang

**Affiliations:** 1 Laboratory for Lipid Medicine and Technology, Department of Medicine, Massachusetts General Hospital and Harvard Medical School, Boston, Massachusetts, United States of America; 2 Laboratory for Genetics, Nutrition and Health, Guangdong Medical College, Zhanjiang, Guangdong, P. R. China; 3 Department of Pharmaceutical Science, North Dakota State University, Fargo, North Dakota, United States of America; 4 State Key Laboratory of Quality Research in Chinese Medicine (UM), Institute of Chinese Medical Sciences, University of Macau, Taipa, Macao SAR, China; Max Delbrueck Center for Molecular Medicine, Germany

## Abstract

Recent studies have shown that a tumor-supportive microenvironment is characterized by high levels of pro-inflammatory and pro-angiogenic eicosanoids derived from omega-6 (n−6) arachidonic acid (AA). Although the metabolic pathways (COX, LOX, and P450) that generate these n−6 AA eicosanoids have been targeted, the role of endogenous AA production in tumorigenesis remains unexplored. Delta-6 desaturase (D6D) is the rate-limiting enzyme responsible for the synthesis of n−6 AA and increased D6D activity can lead to enhanced n−6 AA production. Here, we show that D6D activity is upregulated during melanoma and lung tumor growth and that suppressing D6D activity, either by RNAi knockdown or a specific D6D inhibitor, dramatically reduces tumor growth. Accordingly, the content of AA and AA-derived tumor-promoting metabolites is significantly decreased. Angiogenesis and inflammatory status are also reduced. These results identify D6D as a key factor for tumor growth and as a potential target for cancer therapy and prevention.

## Introduction

The identification of factors that modulate tumorigenesis is crucial for cancer prevention and treatment. Most studies on cancer drug discovery have tried to target cell proliferation using cell- based screening systems to identify anti-cancer compounds; however, the translation of such discoveries into cancer therapy has had limited success [1], [2]. Current research shows that targeting cancer metabolism or factors that modulate the tumor microenvironment may be promising venues for cancer therapy [3], [4].

Arachidonic acid (AA), an omega-6 (n−6) polyunsaturated fatty acid, is converted through three major pathways– the cyclooxygenase (COX), lipoxygenase (LOX), and cytochrome P450 epoxygenase pathways–into bioactive lipid mediator eicosanoids, including prostaglandins (PGs), leukotrienes (LTs), and epoxyeicosatrienoids (EETs), respectively [5], [6] (**Figure S1**). These metabolites have crucial roles in chronic inflammation and cancer [5]–[7]. Increased AA metabolism and eicosanoid formation is a common feature of various types of cancer cells [8], [9]. AA-derived pro-inflammatory eicosanoids, particularly PGE_2_ and LTB_4_ which are produced by tumor cells and their surrounding stromal cells, are key mediators in their crosstalk and can accelerate tumor growth and metastasis through several mechanisms [5], including: 1) directly activating their receptors on tumor cells to induce cell proliferation, survival, migration, and invasion through multiple signaling pathways in both autocrine and paracrine manners, 2) directly inducing cancer cells to secrete growth factors, pro-inflammatory mediators and angiogenic factors that turn a normal microenvironment into one that supports tumor growth and spread, and 3) directly binding receptors on stromal cells to promote a tumor-supportive microenvironment by inducing angiogenesis and evading attack by the immune system [5]. Certain hydroxyeicosatetraenoic acids (HETEs) and EETs derived from AA through the LOX and cytochrome P450 epoxygenase pathways have also been shown to promote angiogenesis, tumor growth and metastasis [10], [11]. Evidently, AA metabolites play a significant role in tumorigenesis, mainly through the promotion of inflammation and angiogenesis.

Given the importance of AA metabolites in cancer biology, many studies have developed anti-cancer drugs targeting the major pathways (COX, LOX, or P450) of AA metabolism [5]. Although some of the drugs targeting eicosanoid signaling, such as aspirin and celecoxib, have shown efficacy in suppressing cancer, they have also been associated with unacceptable toxicity [5], [12]; thus, other strategies must be identified for targeting AA metabolism. Regarding tumorigenesis, the role of endogenous AA production, which occurs upstream of these metabolic pathways (**Figure S1**), remains unexplored.

AA, which is highly abundant in the modern Western diet and in body tissues [13], [14], is synthesized from linoleic acid (LA) through a series of elongation and desaturation enzyme systems. Among these enzymes, delta-6 desaturase (D6D) is the first and rate-limiting step in the production of AA (**Figure S1**). Thus far, the role of D6D in cancer development has not been established. Here, we show that D6D activity is up-regulated during the growth of melanoma and lung tumors and that suppressing D6D activity dramatically reduces tumor growth. Our results suggest that D6D is a key factor for tumor growth, and therefore, it is a potential target for cancer therapy and prevention.

## Methods

### Ethics Statement

All animal procedures were carried out in accordance with the guidelines set by the Massachusetts General Hospital Animal Committee and with IACUC approval (Protocol # 2010N000038). All efforts possible were made to limit animal suffering.

### D6D RNAi Lentivirus Vector and Stable Cell Lines

The Bam HI site of the transfer plasmid pHRST-IRES-GFP was inactivated by blunting the sticky ends with Klenow I and religating with T4 DNA ligase. Then, the RNAi lentivirus vector pLu6-RNAi was obtained by inserting two copies of the hU6 promoter into the Eco RI site of the plasmid. The D6D RNAi lentivirus vector pLu6-RNAi-D6D was obtained by ligating short hairpin mouse D6D oligonucleotides with the large fragment of pLu6-RNAi after Bam HI and Xho I digestion and agarose gel electrophoresis (**Figure S2**). 293T cells (ATCC: CRL-11268, Manassas, VA, authenticated prior to accession by DNA fingerprinting) were transfected with pLu6-RNAi or pLu6-RNAi-D6D transfer plasmid and pHDM-G, pHDM-tat1b, pHDM-Hgpm2, and pRC/CMV-rev1B helper plasmids using SuperFect transfection reagent. Twenty-four and 48 hours after transfection, the lentivirus-containing supernatant was harvested and added to B16-F0 (ATCC: CRL-6322, Manassas, VA) and LLC cells (ATCC: CRL-1642, Manassas, VA) in a media supplemented with 8 µg/ml polybrene. The GFP-positive cells of vector control and D6D-RNAi were sorted on FACSAriaII (BD Biosciences (Billerica, MA) 7 days after virus infection.

### Mouse Tumor Models

Female C57BL6 mice were used for this study. Animals were fed a diet high in n−6 and very low in n−3 fatty acids (**Table S1**) (Mod AIN-76A with safflower oil; Land O’Lakes Purina Feed, LLC, Richmond, IN) until the desired age (10–12 weeks) for experiments was reached. Each mouse was injected subcutaneously into both sides of the lateral abdomen with 2×10^6^ B16-F0 or LLC cells suspended in 50 µl of PBS. To obtain tumor tissues of different sizes, the mice were sacrificed and the tumor tissues were harvested at 2- to 4-day intervals. To investigate the antitumor effects of SC-26196, wild type B16-F0 or LLC cancer cell-bearing mice were intragastrically injected with SC-26196 (100 mg/kg body weight/day; the SC-26196 was kindly provided by the Chemical Synthesis and Drug Supply Program (CSDSP) of the National Institute of Mental Health (NIMH), Bethesda, MD, USA) that was suspended in 0.5% methyl cellulose. Tumor volume, based on caliper measurements, was calculated every day (B16-F0) or every 2 days (LLC) according to the following formula: tumor volume = the shortest diameter^2^× the largest diameter×0.5. After 14 days (B16-F0) or 25 days (LLC) of inoculation, the mice were sacrificed, and the tumor tissues were harvested and stored at −70°C.

### Analysis of Fatty Acids and Eicosanoids

The fatty acid composition of cultured cells and tissues was analyzed by gas chromatography as previously described [15]. For the measurement of eicosanoids**,** a quantity of 50 mg of tumor tissue was homogenized in 2 ml water on ice for 45 s. Methanol and water were added to make the total 3 ml 15% methanol. The homogenate was incubated for 1 h on ice after adding 30 ng D4-PGE_2_ and 300 ng D4-LA (internal standards) and vortexing. The pH was adjusted to 3.0 with 0.2 M HCl. The mixture was loaded on a SPE column preconditioned with 2 ml methanol and 2 ml water. The SPE column was washed with 1 ml water, and the eicosanoids were eluted with 3 ml ethyl acetate. The ethyl acetate solution was dried under nitrogen. The residue was dissolved in 200 µl methanol, and 50 µl were subjected to LC/MS analysis. The Agilent 1200 Series Liquid Chromatography/Mass Selective Detector (LC/MSD) system was used for separation and detection. A gradient chromatographic separation was performed on a ZORBAX Eclipse XDB-C18 column (Agilent, 5 µm, 75×4.6 mm) at 25°C. The detection was made in the negative mode. D4-PGE2 was used as an internal standard for quantification of all eicosanoids. The concentrations of eicosanoids in the samples were calculated by comparing their ratios of the peak areas of the compounds to the internal standards (**Figure S3**).

### Angiogenesis Assay

Microvessel density in tumors was determined by immunohistological staining using an anti-CD31 antibody and expressed as a percentage of CD31 stained area per section, as described previously [16]. An *in vivo* angiogenesis assay was performed as described by Ito et al. [17] with the following modifications. Geltrex™ Basement Membrane Matrix 0.4 mL premixed with bFGF (5 µg/ml) with and without 100 µM SC-26196 was injected subcutaneously into C57BL6 mice (3 mice/group). Mice were sacrificed 7 days after injection and dissected to expose the implants for recording.

### Statistical Analysis

Comparisons were made between the 2 treatments using Student’s *t* tests. Differences in tumor growth rate between the control and treatment (D6D-RNAi or D6D inhibitor) groups or between B16 melanoma and LLC lung cancer groups was evaluated by two-way repeated measures ANOVA analyses followed by Bonferroni tests. Linear regression analysis was used for determining the significance of associations between log-transformed tumor size and D6D activity or expression. Differences were considered significant at the level of *P*<0.05. Statistical analysis was performed using GraphPad Prism 5 (La Jolla, CA).

## Results

### D6D Activity is Higher in Tumor Tissue than in Adjacent Non-tumor Tissue

**Figure 1:**
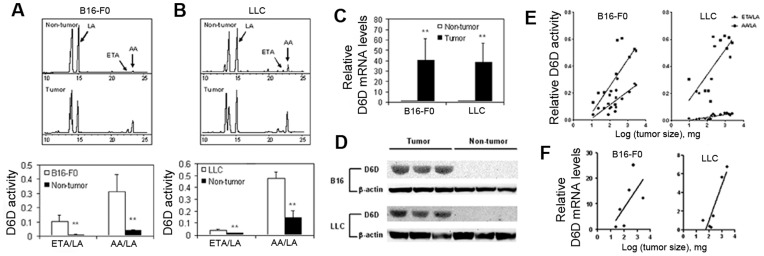
D6D activity is higher in tumor tissue than in adjacent non-tumor tissue. (**a**) The activity of biomarkers of D6D in B16 melanoma. *Upper:* Gas chromatography showing the differences in LA, ETA and AA content between B16 melanoma and adjacent non-tumor tissue. *Lower*: The ratios of ETA/LA and AA/LA indicate D6D enzyme activity. **P<0.01, n = 4. (**b**) Activity of biomarkers of D6D in LLC tumors. *Upper:* Gas chromatography showing the differences in LA, ETA and AA content between LLC tumor and adjacent non-tumor tissue. *Lower*: The ratios of ETA/LA and AA/LA indicate D6D enzyme activity. **P<0.01, n = 4. (**c**) D6D mRNA levels in tumor and adjacent non-tumor tissue. **P<0.01, n = 3. (**d**) Western blot showing D6D protein levels in tumor and adjacent non-tumor tissue. (**e**) Correlation of the activity of biomarkers of D6D (based on ETA/LA and AA/LA ratios) with tumor size. (**f**) Correlation of D6D mRNA levels with tumor size.

**Figure 2:**
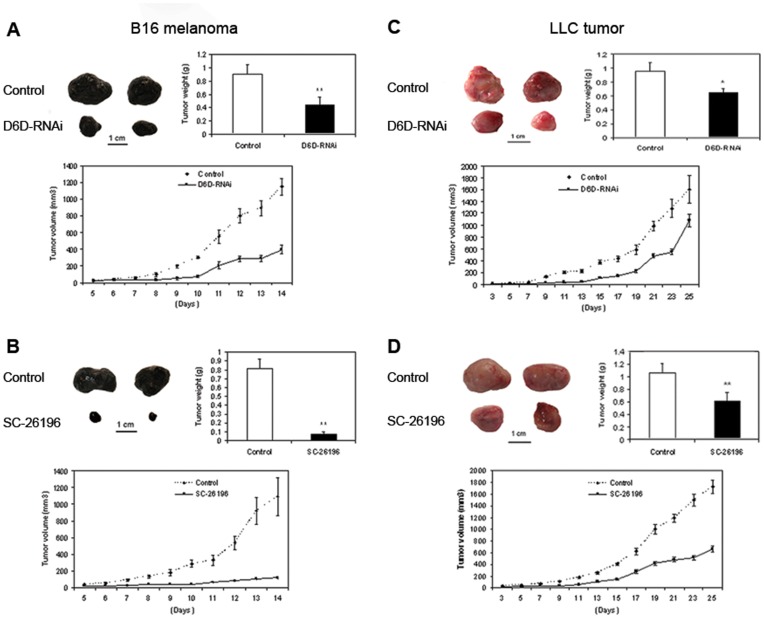
Reducing D6D expression or activity suppresses tumor growth. (**a**) Effect of D6D-RNAi knockdown on B16 melanoma growth. **P<0.001; n = 8 for both groups. (**b**) Effect of the D6D selective inhibitor SC-26196 on B16 melanoma growth. **P<0.0001; control, n = 7; treated, n = 8. (**c**) Effect of D6D-RNAi knockdown on LLC tumor growth. *P<0.05; n = 8 for both groups. (**d**) Effect of the D6D selective inhibitor SC-26196 on LLC tumor growth. **P<0.01; control, n = 7; treated, n = 10. (**a–d**) *Upper left*: Representative tumor sizes. *Upper right*: Average tumor weight at the end of the experiment. *Lower*: Tumor growth rate during the 14-day experimental period.

To determine whether there is a difference in D6D activity between tumor and non-tumor tissues, we analyzed the activity of biomarkers of the D6D enzyme and expression levels in tumor and non-tumor tissues from B16 melanoma and Lewis lung cancer (LLC) tumors implanted in C57B6 mice. Since D6D catalyzes the first rate-limiting step of the enzymatic conversion of LA to AA, with the formation of eicosatrienoic acid (ETA) as an intermediate product, the ratios of ETA to LA and AA to LA reflect the activity of the biomarkers of D6D [18]. The results show that the tumor tissue content of AA was 4 times greater for B16 melanoma (**Figure 1A**) and 2 times greater for LLC tumors (**Figure 1B**), compared to adjacent non-tumor tissues. Accordingly, the ETA/LA and AA/LA ratios were significantly higher in both tumor tissues than in their adjacent non-tumor tissues (**Figures 1A, 1B**). To determine whether the higher D6D activity observed was due to higher expression of D6D, we measured D6D mRNA and protein levels. As seen in **Figure 1**, the D6D mRNA expression levels were 40 times higher in tumor tissues than in adjacent non-tumor tissues (**Figure 1C**) whereas D6D protein was abundantly expressed in tumor tissues but almost undetectable in adjacent normal tissues (**Figure 1D**). These data indicate that D6D is up-regulated in tumor tissue.

We also examined whether there was a correlation between tumor size and D6D activity. The activity of the biomarkers of D6D was analyzed in B16 and LLC tumors of different sizes. As shown in **Figure 1E**, the ETA/LA and AA/LA ratios were positively correlated with the sizes of both the B16 and LLC tumors (ETA/LA in B16 melanoma: *P* = 0.0015, *r* ^2^ = 0.58; AA/LA in B16 melanoma: *P* = 0.0025, *r* ^2^ = 0.55; ETA/LA in LLC tumors: *P* = 0.0001, *r* ^2^ = 0.70; AA/LA in LLC tumors: *P* = 0.0017, *r* ^2^ = 0.54). D6D mRNA expression levels were also correlated with tumor size in the B16 melanoma (*P* = 0.0017, *r* ^2^ = 0.93) and LLC tumors (*P* = 0.0139, *r* ^2^ = 0.81) (**Figure 1F**). These results suggest a positive correlation between the activity of the biomarkers of D6D and tumor growth.

### Reducing D6D Activity Suppresses Tumor Growth

To examine the role of D6D in tumorigenesis, we reduced D6D activity either by knocking down D6D expression with RNAi or by inhibiting D6D enzyme activity with SC-26196, a highly selective inhibitor of D6D [19]. We then measured the growth rate of the B16 melanoma and LLC tumors in mice. Our data indicate that treatment with RNAi or SC-26196 can effectively reduce D6D activity (**Figures S4, S5 and S6**).

D6D-RNAi or the D6D selective inhibitor SC-26196 remarkably suppressed the growth of the B16 melanoma and LLC tumors (**Figure 2**). For the B16 melanoma model, all control groups of mice developed palpable B16 melanoma tumors by day 7 whereas the treatment groups (D6D-RNAi or SC-26196) did not develop palpable tumors until days 10 (**Figure 2A**) and 12 (**Figure 2B**), respectively, over an observation period of 14 days. The tumor growth rate of B16 melanoma treated with D6D-RNAi or SC-26196 was much slower than that of the control group (**Figures 2A, 2B**). The percent inhibition of B16 melanoma growth (based on tumor weight after harvest) by D6D-RNAi and SC-26196 was 53±13% (*P*<0.01) and 91±6% (*P*<0.01), respectively. The mice treated with SC-26196 showed no signs of significant toxicity, which may include reduced mobility, reduced body weight, piloerection, hunched-back posture, anorexia, diarrhea, or somnolence.

Similarly, for the LLC tumor model, all control groups of mice developed palpable tumors by day 7 whereas the treatment groups (SC-26196 or D6D-RNAi) developed palpable tumors by days 13 (**Figure 2C**) and 15 (**Figure 2D**), respectively, over an observation period of 25 days. The growth rates of LLC tumors treated with D6D-RNAi or SC-26196 were both significantly slower when compared to that of the control groups (**Figures 2C, 2D**). The percent inhibition of LLC tumor growth (based on tumor weight after harvest) by D6D-RNAi and SC-26196 were 32±6% (*P*<0.05) and 42±5% (*P*<0.01), respectively. These data indicate that reduction of D6D activity is highly effective in suppressing tumor growth, especially for B16 melanoma.

### Decreased D6D Reduces the Levels of AA and AA-derived Eicosanoids in Tumor Tissue

**Figure 3:**
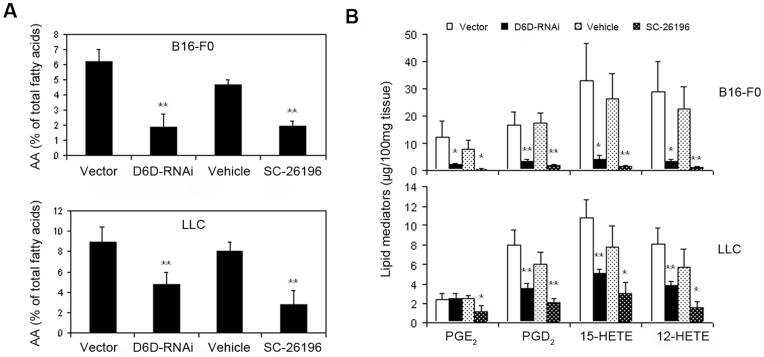
Decreased D6D reduces the levels of AA and AA-derived eicosanoids in tumor tissue. Knocking down D6D (with RNAi) or inhibiting D6D (with selective inhibitor SC-26196) decreased (**a**) AA and (**b**) AA metabolite levels in mouse models of B16 melanoma and LLC. *P<0.05; **P<0.01; n = 3–5.

To test whether decreased D6D results in reduced production of AA and its metabolites in tumor tissue, we measured the levels of AA and AA-derived eicosanoids in tumor tissues by gas chromatography (GC) and liquid chromatography-mass spectrometry (LC-MS). As shown in **Figure 3A** **and** **Table 1**, the tumor content of AA decreased by 47–69% in D6D-RNAi- or SC-26196-treated B16 melanoma and LLC tumors as compared to the control groups. The levels of AA-derived eicosanoids (including PGD_2_, PGE_2_, 12-HETE, and 15-HETE) decreased by 80–95% in D6D-RNAi- or SC-26196-treated B16 melanoma compared to the control groups (**Figure 3B**). A significant reduction of AA-derived eicosanoids was also observed in D6D-RNAi- and SC-26196-treated LLC tumors; however, the degree of inhibition was lower compared with that observed in B16 melanoma. These results are consistent with the inhibitory effects of reduced D6D on tumor growth in the treated B16 and LLC tumors.

**Table 1:** Fatty acid composition of B16 and LLC tumor tissues (% of total fatty acids).

		C18∶1 (OA)	C18∶2n6(LA)	C18∶3n3 (ALA)	C20∶3n6 (ETA)	C20∶4n6(AA)	C20∶5n3 (EPA)	C22∶4n6 (DTA)	C22∶5n6 (DPAn6)	C22∶5n6 (DPAn3)	C22∶6n6 (DHA)
**B16**	Vehicle	24.58±1.55	16.41±4.31	0.12±0.04	1.54±0.16	4.66±0.32	UD	0.64±0.06	0.03±0.04	0.12±0.01	0.37±0.04
	SC-26196	27.3±2.24	18.53±2.81	0.13±0.02	1.01±0.28^a^	1.94±0.31^b^	UD	0.27±0.03^b^	UD	UD	0.04±0.07^b^
	Vector	24.05±1.63	12.63±1.48	0.14±0.02	1.71±0.24	6.18±0.67	0.18±0.04	0.66±0.09	UD	0.17±0.08	0.31±0.14
	RNAi	30.28±3.21^a^	19.75±1.42^b^	0.11±0.05^b^	0.88±0.29^b^	2.01±0.64^b^	0.12±0.09	0.06±0.1^b^	UD	UD	0.07±0.09^b^
**LLC**	Vehicle	11.82±1.18	15.25±1.23	0.9±0.35	0.67±0.09	7.98±0.78	0.09±0.04	2.94±0.54	0.79±0.47	0.55±0.09	2.11±0.31
	SC-26196	19.18±1.42^b^	25.54±3.98^a^	1.02±0.76	0.43±0.29	2.76±1.17^b^	0.03±0.01^a^	0.79±0.32^b^	0.16±0.08	0.28±0.13	0.56±0.25^b^
	Vector	13.95±1.95	16.59±2.22	1.2±0.46	0.68±0.13	8.95±1.47	UD	4.51±0.58	UD	0.25±0.36	1.35±0.39
	RNAi	21.28±3.81^b^	21.39±3.18^b^	2.29±0.77^a^	0.42±0.14^b^	4.73±1.17^b^	UD	2.46±0.44^b^	UD	UD	0.55±0.18^b^

The data are expressed as means ± SD. UD: undetectable.

a *P*<0.05,

b *P*<0.01, compared to vehicle or vector control. Student’s *t*-test.

### Decreased D6D Suppresses Tumor Angiogenesis and Inflammation

**Figure 4:**
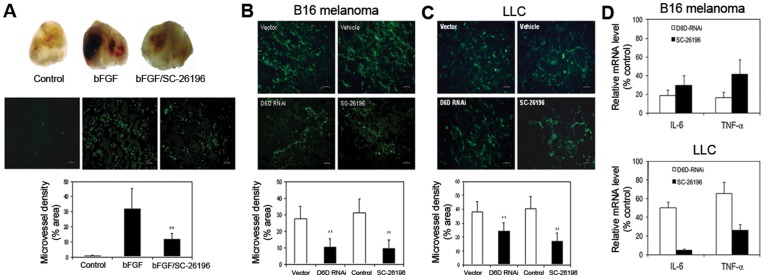
Decreased D6D suppresses tumor angiogenesis and inflammation. (**a**) SC-26196, a D6D selective inhibitor, at 100 µM significantly inhibited basic fibroblast growth factor (bFGF)-induced angiogenesis in a Matrigel plug assay (**P<0.01; n = 3). (**b, c**) Immunohistological staining of microvessel density in tumors. Knocking down D6D (with RNAi) or inhibiting D6D (with selective inhibitor SC-26196) reduced angiogenesis in (**b**) B16 melanoma and (**c**) LLC tumors (**P<0.01; n = 3). (**d**) The mRNA expression of the inflammatory factors interleukin-6 (IL-6) and TNF-α is down-regulated in B16 melanoma and LLC lung tumors treated with D6D-RNAi or SC-26196, shown here by percent of control. **P<0.01; n = 4.

Because D6D inhibition does not seem to significantly affect cell proliferation in *in vitro* models (**Figure S7**), we suspect that the anti-tumor effects of D6D inhibition may be due to its influence on characteristics of the tumor microenvironment, such as angiogenesis and inflammation. Therefore, we examined whether decreased D6D affects the status of tumor angiogenesis and inflammation. The effects of D6D inhibition on angiogenesis status were measured by both a Matrigel plug assay and immunohistological staining (IHS). As shown in **Figure 4A**, SC-26196 at 100 µM significantly inhibited basic fibroblast growth factor (bFGF)-induced angiogenesis in a Matrigel plug assay by 64% (*P*<0.01). Furthermore, the angiogenesis evaluated by IHS was significantly decreased in D6D-RNAi- or SC-26196-treated B16 melanoma (**Figure 4B**) and LLC tumors (**Figure 4C**) compared to their respective control groups (*P*<0.01). The inhibitory effects on angiogenesis in treated B16 melanoma were stronger than those in treated LLC tumors. The inflammation status in B16 melanoma and LLC tumors with and without D6D inhibition was determined by measuring gene expression of the inflammatory cytokines IL-6 and TNF-alpha. As shown in **Figure 4D**, knocking down or inhibiting D6D reduced mRNA expression of IL-6 and TNF-alpha expression in B16 and LLC tumors, suggesting a reduced inflammatory state in the treated tumors. The results support an anti-angiogenic and anti-inflammatory effect of D6D inhibition.

## Discussion

Tumor initiation and progression require a tumor-supporting microenvironment, such as inflammation and angiogenesis [4], [5]. Metabolites of AA have long been known to be critical pro-angiogenic and pro-inflammatory mediators [5]. Therefore, inhibitors of cyclooxygenase such as COX-2 have been a subject of intense research, and COX-2 has been well recognized as an anti-cancer therapy target [5], [8], [9]. However, there is little information on whether the endogenous synthesis of AA affects eicosanoid production and tumorigenesis. In this study, we show that D6D, the rate-limiting enzyme for AA synthesis, is up-regulated in tumor tissues and that suppression of its expression or activity results in a remarkable reduction of tumor growth associated with a decrease in AA-derived eicosanoids, angiogenesis, and inflammation in tumor tissues. These results demonstrate that D6D is a potentially critical factor for tumorigenesis and that targeting this enzyme is effective in suppressing tumor growth.

D6D catalyzes the first and rate-limiting step of the conversion of essential fatty acids, which is converting n−6 linoleic acid (LA, 18∶2n−6) and n−3 α-linolenic acid (ALA, 18:n−3) to the longer chain polyunsaturated fatty acids (LC-PUFA) arachidonic acid (AA, 20∶4n−6) and eicosapentaenoic acid (EPA, 20∶5n−3), respectively. These two classes of PUFA compete with each other for the same metabolizing enzymes but their metabolites are functionally distinct and often have opposing physiological effects [6], [7], [20]. For example, the n−6 PUFA-derived metabolites promote inflammation and angiogenesis, whereas those derived from n−3 PUFA have anti-inflammatory and anti-angiogenic properties [7], [21], [22]. Although D6D prefers ALA as a substrate [23], LA (the n−6 substrate of D6D) is much more abundant in the modern Western diet and body tissues than ALA (the n−3 substrate of D6D), with an n−6/n−3 fatty acid ratio of >10∶1 [13], [14]. Therefore, high levels of endogenous AA are synthesized and contribute to eicosanoid production. It has been suggested that endogenous and exogenous AA are metabolized differently, with more endogenous AA being metabolized into pro-inflammatory eicosanoids [24]. Thus, increased D6D predominantly favors the LA-AA pathway and greatly increases the amount of endogenous AA available for the production of pro-cancer eicosanoids.

Our findings that the expression and activity of the biomarkers of D6D and the levels of AA are remarkably higher in tumor than adjacent normal tissues, as well as our observation that tumor size is positively correlated with higher activity of the biomarkers of D6D activity, suggest that D6D plays a key role in tumorigenesis. Our consistent results in the increased expression and functional activity of D6D in two different cancer types (melanoma and lung cancer) point to the possibility that up-regulation of D6D is a common characteristic of malignant tumors. This possibility is supported by previous studies in humans using microarray analysis that have shown a significant difference in D6D mRNA between tumor and normal tissues (>2-fold higher in tumors than in normal tissue) from patients with breast cancer or brain tumors [25], [26]. Nevertheless, further study is warranted to examine the D6D status of human tumor samples of various cancer types. We noticed that there was a considerable amount of AA in adjacent normal tissues in the context of almost undetectable D6D expression. We assumed that this AA was synthesized in other tissues or organs (e.g., the liver) and transferred through the circulation system. The mechanisms by which D6D is up-regulated during tumor development remain to be explored. We speculate that D6D is up-regulated by increased activity of SREBP-1c or PPARα in a tumor microenvironment characterized by hypoxia and aberrant energy metabolism. Previous studies have shown that D6D expression may be regulated by SREBP-1c [27] and that SREBP-1c can be stimulated under hypoxic conditions via hypoxia inducible factor-1alpha (HIF-1α) [28]. In addition, SREBP-1c is the key factor for up-regulating fatty acid synthesis in many cancers [29]. Given the fact that hypoxia and increased ROS are common conditions in the tumor microenvironment, it is possible that D6D expression in tumors results from the following pathway: Hypoxia/ROS → HIF-1α → SREBP-1c → D6D. Thus, the discovery of increased D6D activity in tumors opens up a new field of investigation in cancer biology. Our findings suggest that increased expression and activity of D6D is potentially a new cancer biomarker.

Our results showing that inhibition of D6D activity, by either RNAi-knockdown or a selective inhibitor, is highly effective in suppressing tumor growth indicate that D6D is a target for cancer therapy. Given that D6D is the rate-limiting enzyme for AA synthesis and that LA is the major substrate for generating AA in our bodies, it is conceivable that the activity of D6D is critical for the production of AA and AA-derived pro-cancer eicosanoids and thereby affects tumorigenesis. From knowledge of these biochemical pathways, it is logical to speculate that D6D inhibition was effective in this study due to its inhibition of AA and its metabolites. D6D inhibition, which has a target upstream of AA metabolism, is more efficient in reducing pro-cancer eicosanoid production compared to the inhibition of individual pathways such as COX, LOX, or P450 (by which pro-cancer AA metabolites form), thus making D6D inhibition potentially more effective in suppressing cancer development. Thus far, therapeutic agents that target AA production/D6D activity do not exist, so the development of such drugs may provide effective therapeutic options, as demonstrated in the present study by the high efficacy of SC-26196, a selective D6D inhibitor. This idea also supported by a previous study showing that SC-26196 impeded intestinal tumorigenesis by inhibiting the synthesis of AA [30].

During treatment with SC-26196, we did not observe any signs of toxicity in the mice such as reduced mobility, piloerection, hunched-back posture, etc. In addition, D6D-RNAi and SC-26196 at concentrations of up to 128 µM did not show significant growth suppression in both B16 and LLC cells *in vitro* (**Figure S7**), suggesting that blocking D6D is not directly toxic to cancer cells. However, cell proliferation is not necessarily related to tumor growth and is probably not an accurate measure of the effects of D6D inhibition on cancer. Rather, the anti-tumor effect of D6D inhibition is likely due to its impact on the tumor microenvironment, such as angiogenesis and inflammation, which are the key factors that facilitate tumor growth and survival [4], [5]. It is well documented that AA-derived eicosanoids play a central role in inflammation [5]–[7], [31]. Inflammatory cells (e.g., macrophages, neutrophils, natural killer cells, etc.) and factors (e.g., PGE2, matrix metallopeptidases, interleukins, chemokines, etc.) are well-known to induce angiogenesis [32]–[34]. A growing body of evidence has demonstrated that AA-derived eicosanoids stimulate angiogenesis by up-regulating angiogenic factors. PGE2 stimulates angiogenesis by activating the fibroblast growth factor receptor (FGFR), epidermal growth factor receptor (EGFR) and β3 integrin through E prostanoid 2 (EP2)- and EP4-mediated pathways [5], [35], [36]. Lipoxygenase metabolites, including 12-HETE and 15-HETE, have also been reported to promote angiogenesis by inducing FGF [37], interleukin-8 (IL-8, a macrophage-derived mediator of angiogenesis) [38], and vascular endothelial growth factor (VEGF) [39]. Our results revealed that the pro-angiogenic, AA-derived eicosanoids PGE_2_, 12-HETE, and 15-HETE, as well as the key inflammatory factors interleukin-6 (IL-6) and tumor necrosis factor α (TNF-α), were dramatically reduced in B16 melanoma and LLC tumors treated with D6D-RNAi or SC-26196 (**Figures 3B** **,** **4D**). This suggests that blocking D6D can inhibit inflammation and angiogenesis. Indeed, a remarkable reduction in tumor angiogenesis, as evaluated by immunohistological staining and an *in vivo* angiogenesis model, was observed in the tumors treated with D6D-RNAi or SC-26196 (**Figures 4A–C**). Altogether, our results demonstrate that inhibition of D6D can diminish the production of AA and AA-derived eicosanoids, leading to the reduction of inflammation and angiogenesis and consequently, the suppression of tumor growth.

A potential problem is that D6D inhibition may cause a deficiency of long chain n−6 and n−3 fatty acids (i.e., AA and EPA) in normal tissue. However, because the content of AA in the modern Western diet and body tissues is very high (and even excessive for most people), D6D inhibition would not deplete all AA but simply reduce its endogenous production. In fact, our animals treated with the D6D inhibitor showed no significant side effects. Regarding the long chain n−3 fatty acids (i.e., EPA, DHA), these are mainly derived from specific foods (fish and fish oils), and dietary supplementation of the n−3 fatty acids can readily prevent a deficiency. Given the capability of n−3 fatty acids to compete with n−6 fatty acids for metabolism, thereby reducing AA-derived eicosanoids and their anti-cancer properties, the supplementation of n−3 fatty acids together with the use of D6D inhibitors might not only make D6D inhibition safer, but also enhance its anti-cancer efficacy. As the exogenous AA from animal products may still interfere with the outcome of D6D-inhibition therapy to some extent, the consumption of AA-containing products must be restricted during the treatment. Still, we acknowledge that AA and its metabolites are naturally occurring and essential factors in maintaining normal functioning of the immune system, heart, brain, testes, etc. At present, it is unknown whether there are detrimental effects of significantly reduced AA content due to D6D inhibition, and how long-term use of a D6D inhibitor might impact health. These are important issues that will need to be carefully addressed in future studies.

Our discovery of D6D’s role in tumorigenesis also points to the importance of tissue n−6 and n−3 fatty acid status in cancer prevention. Because of the competition between n−6 and n−3 fatty acids for D6D and other metabolizing enzymes, the relative amounts, or the ratio, of these two classes of fatty acids may be a key factor for tumorigenesis. The severe imbalance between n−6 and n−3 fatty acids (n−6/n−3>10) in the modern Western diet and human body tissue has been thought to contribute to today’s increased risk of diseases, including cancer [14], [18]. Evidence from recent studies using the transgenic *fat-1* mouse model, which can endogenously convert n−6 to n−3 fatty acids and has a balanced n−6/n−3 ratio in its body tissues [40], strongly supports this notion [41]–[48]. These studies have demonstrated that decreasing the tissue ratio of n−6/n−3 can significantly reduce the formation and growth of various cancers, which is associated with reduced levels of cancer-related eicosanoids and genes [41]–[48], suggesting that the tumor**-**promoting effect of increased D6D activity can be diminished by decreasing tissue n−6/n−3 fatty acid ratio. While further study is warranted to explore this exciting field, the results of the present study increase our understanding of the importance of D6D as well as that of the tissue ratio of n−6/n−3 in cancer prevention and also point to a potentially safe and effective approach to cancer prevention through balancing dietary intake of n−6/n−3.

In summary, the results presented here demonstrate an important role for the D6D enzyme in tumorigenesis and reveal a link between lipid metabolism and cancer biology. D6D may serve as a new cancer biomarker and a potential target for the development of novel anti-cancer drugs. The results also highlight the potential utility of n−3 fatty acid supplementation and balancing the tissue ratio of n−6/n−3 fatty acids for cancer prevention and treatment.

## Supporting Information

Figure S1 **Metabolism of polyunsaturated fatty acids.** The long-chain polyunsaturated fatty acids n−6 arachidonic acid (AA) and n−3 eicosapentaenoic acid (EPA) are derived from LA and ALA through a series of desaturation and chain-elongation enzyme systems and are metabolized through the three major pathways cyclooxygenase (COX), lipoxygenase (LOX), and cytochrome P450 epoxygenase. Please note that delta-6 desaturase (D6D) is the rate-limiting enzyme for the synthesis of both n−6 AA and n−3 EPA. Furthermore, the two classes of fatty acids (n−6 and n−3) compete for the same enzymes for both synthesis and metabolism.
(TIF)

Figure S2 **The schematic structure of the pLu6-RNAi-D6D plasmid.**
(TIF)

Figure S3 **Identification and quantification of AA-derived eicosanoids were determined by LC- MS.** A gradient chromatographic separation was performed on a ZORBAX Eclipse XDB-C18 column (Agilent, 5 µm, 75×4.6 mm) at 25°C. The detection was made in the negative mode. D4-PGE2 was used as an internal standard for quantification of all eicosanoids. The concentrations of eicosanoids in the samples were calculated by comparing their ratios of peak areas of compounds to the internal standards.
(TIF)

Figure S4 **D6D mRNA expression in B16 and LLC cells treated with D6D-RNAi** ***in vitro*** **.**
(TIF)

Figure S5 **D6D activity in B16 and LLC cells treated with D6D-RNAi** ***in vitro*** **.**
(TIF)

Figure S6 **Gas chromatography showing the differences in LA, ETA and AA content in treated (with D6D-RNAi or SC-26196) or non-treated tumors.**
(TIF)

Figure S7 **Viability of B16 and LLC cells treated with D6D-RNAi and SC-26196** ***in vitro*** **.** ***P*** **>0.05; n = 4.**
(TIF)

Table S1 Dietary fatty acid composition for C57BL6 mice.
(TIF)
