# Supplementary figures and images for: Inhibiting Delta-6 Desaturase Activity Suppresses Tumor Growth in Mice
Source: PLoS One. 2012 Oct 24;7(10):e47567. doi: 10.1371/journal.pone.0047567 (PMC3480421; doi:10.1371/journal.pone.0047567)

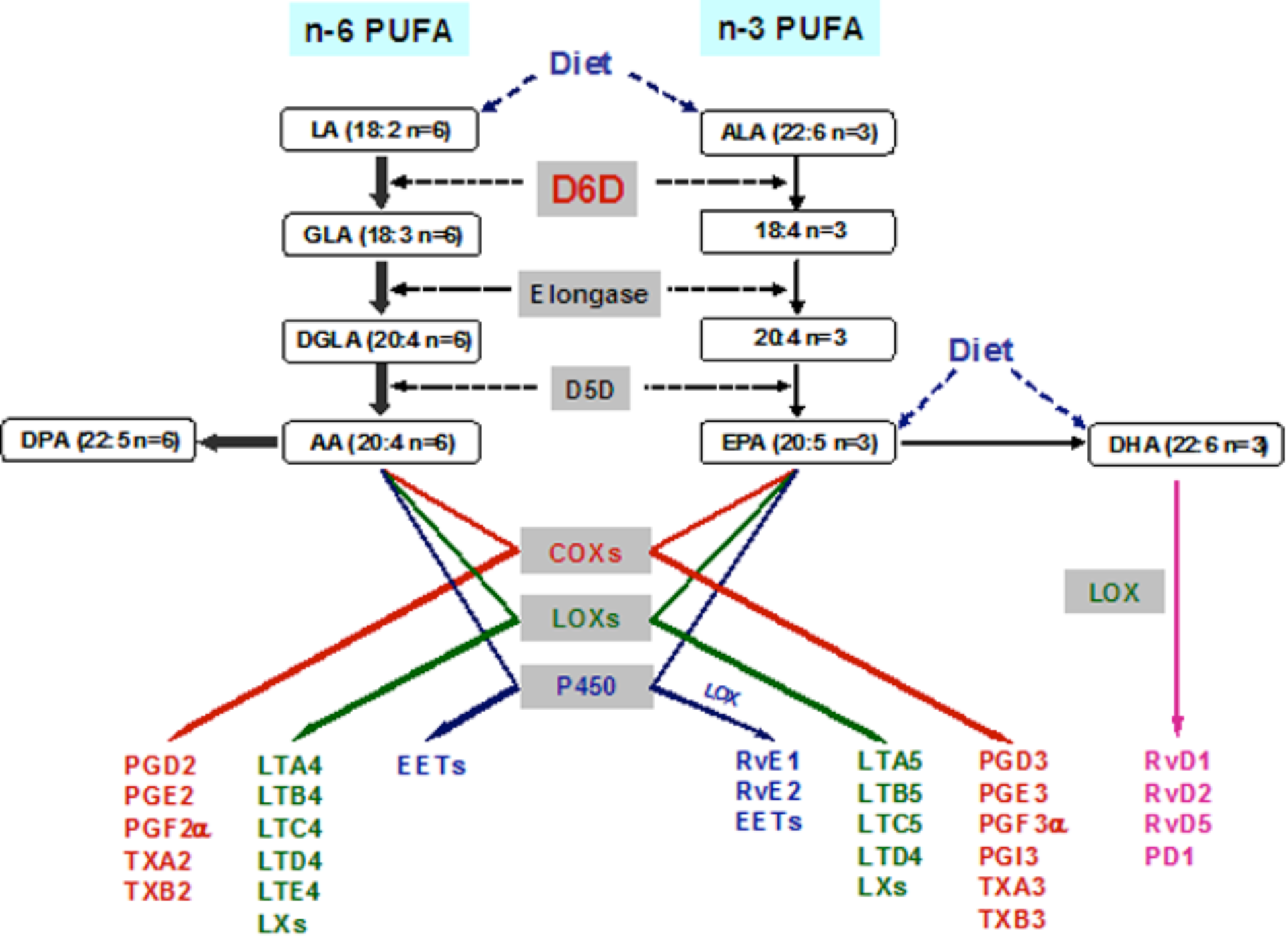

Supplement: Figure S1 — Metabolism of polyunsaturated fatty acids. The long-chain polyunsaturated fatty acids n−6 arachidonic acid (AA) and n−3 eicosapentaenoic acid (EPA) are derived from LA and ALA through a series of desaturation and chain-elongation enzyme systems and are metabolized through the three major pathways cyclooxygenase (COX), lipoxygenase (LOX), and cytochrome P450 epoxygenase. Please note that delta-6 desaturase (D6D) is the rate-limiting enzyme for the synthesis of both n−6 AA and n−3 EPA. Furthermore, the two classes of fatty acids (n−6 and n−3) compete for the same enzymes for both synthesis and metabolism. (TIF) [file pone.0047567.s001.tif]

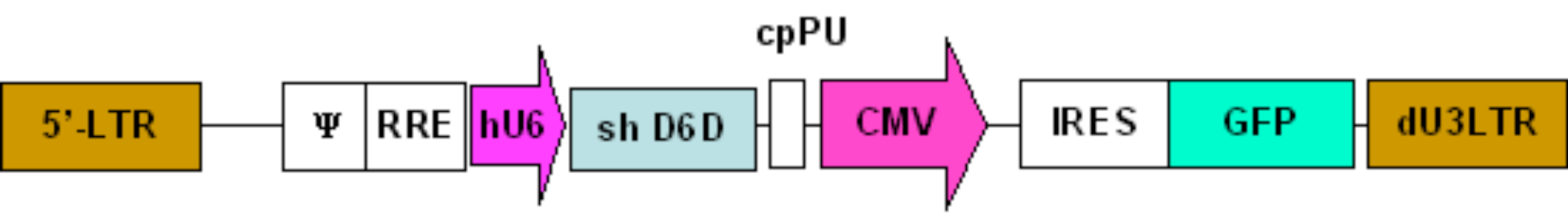

Supplement: Figure S2 — The schematic structure of the pLu6-RNAi-D6D plasmid. (TIF) [file pone.0047567.s002.tif]

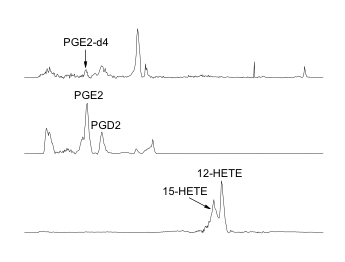

Supplement: Figure S3 — Identification and quantification of AA-derived eicosanoids were determined by LC- MS. A gradient chromatographic separation was performed on a ZORBAX Eclipse XDB-C18 column (Agilent, 5 µm, 75×4.6 mm) at 25°C. The detection was made in the negative mode. D4-PGE2 was used as an internal standard for quantification of all eicosanoids. The concentrations of eicosanoids in the samples were calculated by comparing their ratios of peak areas of compounds to the internal standards. (TIF) [file pone.0047567.s003.tif]

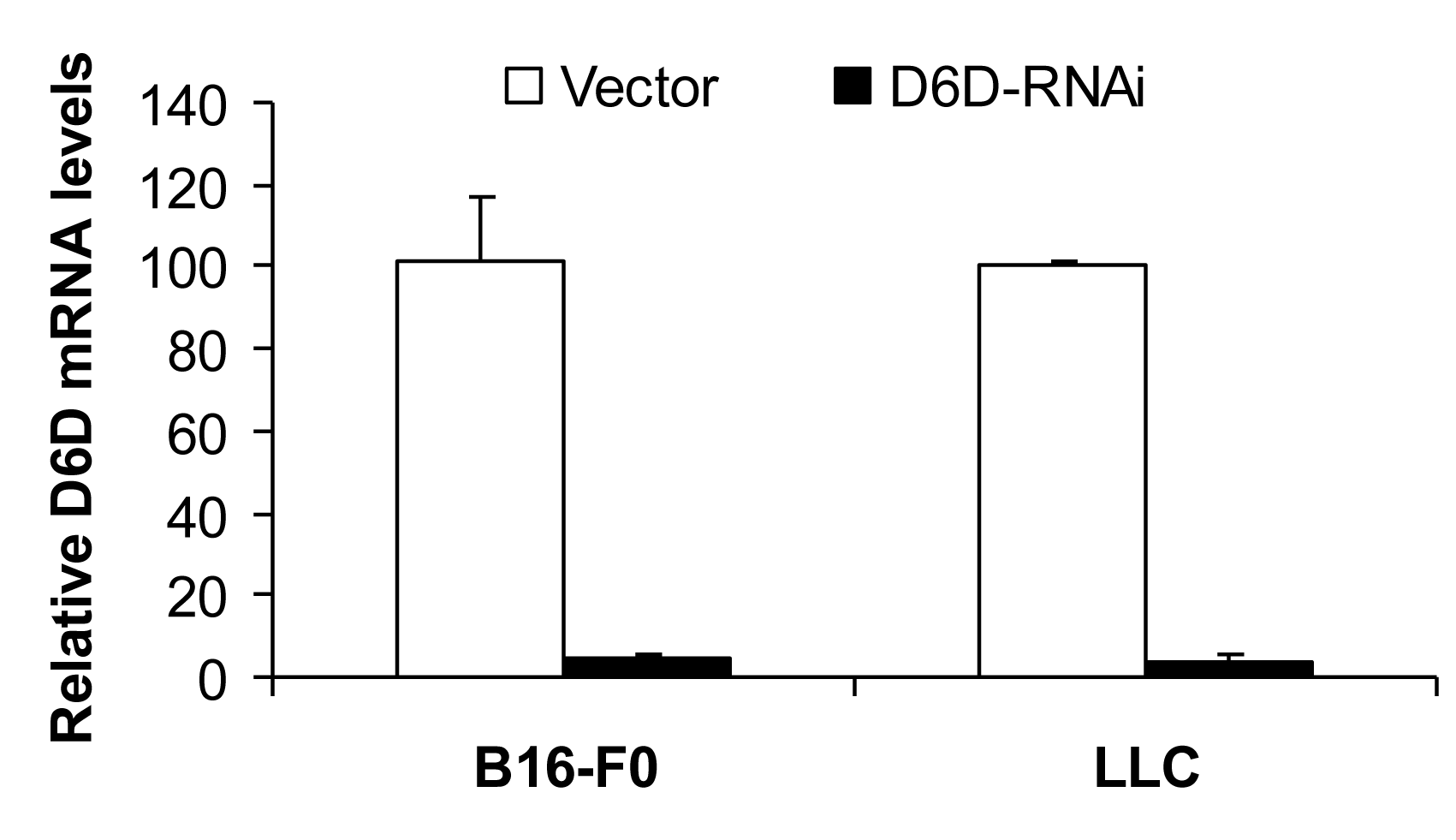

Supplement: Figure S4 — D6D mRNA expression in B16 and LLC cells treated with D6D-RNAi in vitro . (TIF) [file pone.0047567.s004.tif]

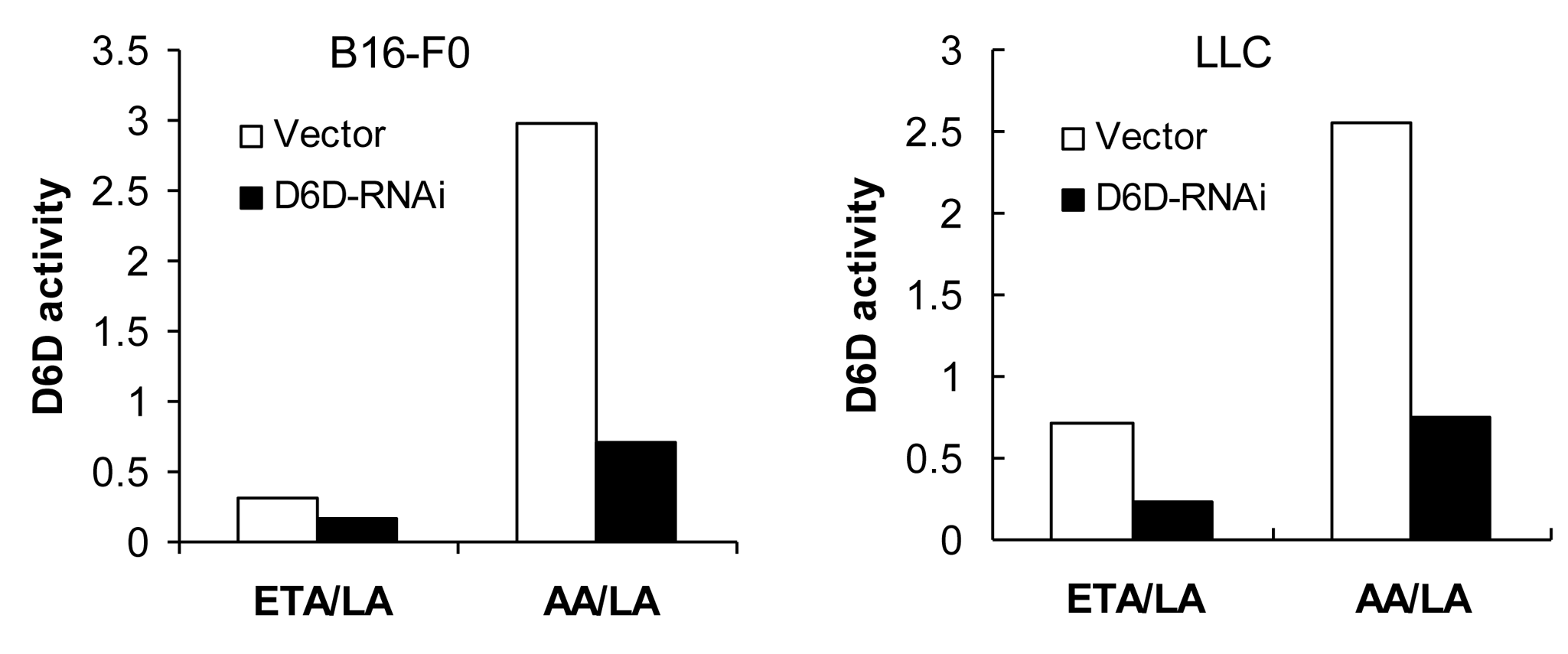

Supplement: Figure S5 — D6D activity in B16 and LLC cells treated with D6D-RNAi in vitro . (TIF) [file pone.0047567.s005.tif]

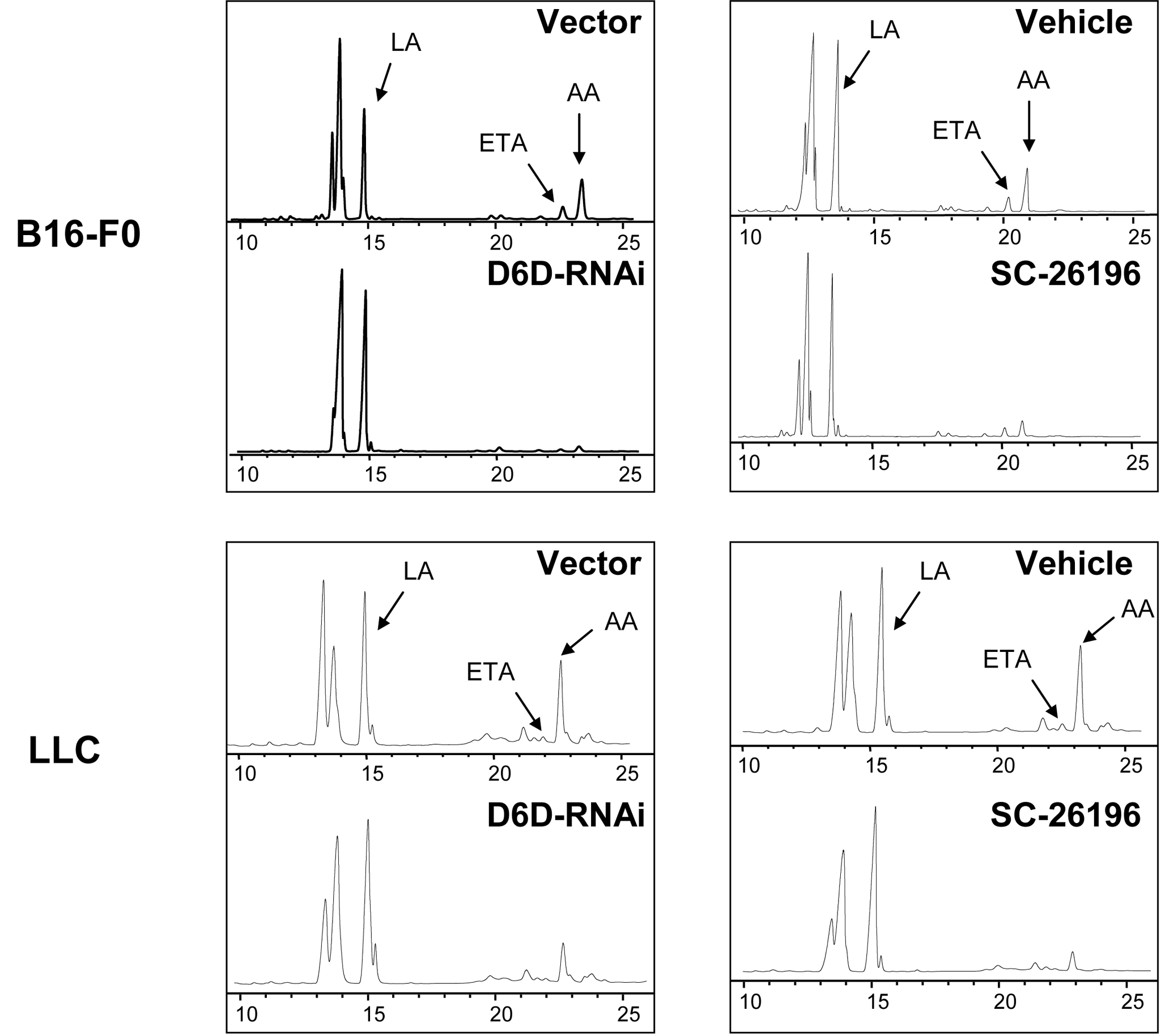

Supplement: Figure S6 — Gas chromatography showing the differences in LA, ETA and AA content in treated (with D6D-RNAi or SC-26196) or non-treated tumors. (TIF) [file pone.0047567.s006.tif]

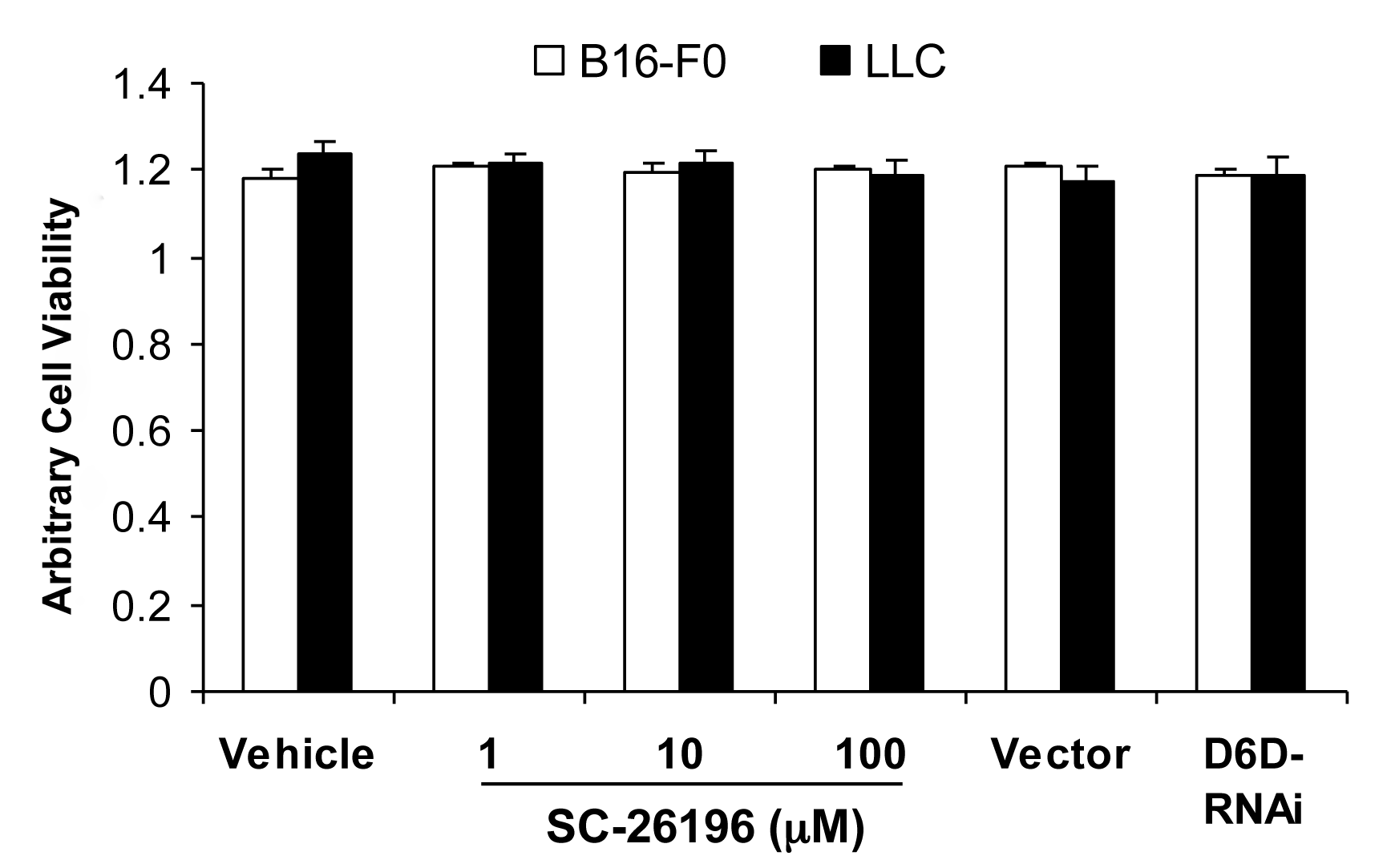

Supplement: Figure S7 — Viability of B16 and LLC cells treated with D6D-RNAi and SC-26196 in vitro . P >0.05; n = 4. (TIF) [file pone.0047567.s007.tif]

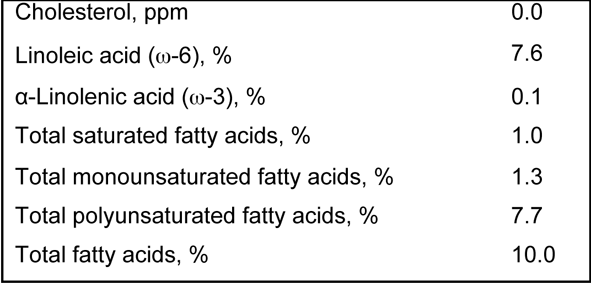

Supplement: Table S1 — Dietary fatty acid composition for C57BL6 mice. (TIF) [file pone.0047567.s008.tif]
